# [Retracted] Germacrone cooperates with dexmedetomidine to alleviate high‑fat diet‑induced type 2 diabetes mellitus via upregulating AMPKα1 expression

**DOI:** 10.3892/etm.2024.12406

**Published:** 2024-01-29

**Authors:** Yang Sun, Lanlan Li, Jun Wu, Bing Gong, Haiyan Liu

Exp Ther Med 18:3514–3524, 2019; DOI: 10.3892/etm.2019.7990

Following the publication of the above paper, it was drawn to the Editors’ attention by a concerned reader that certain of the Masson-stained images shown in Fig. 2A and the TUNEL assay data shown in Figs. 2B and 5G were strikingly similar to data appearing in different form in other articles written by different authors at different research institutes which had either already been published elsewhere prior to the submission of this paper to *Experimental and Therapeutic Medicine*, or were under consideration for publication at around the same time (some articles of which have been retracted). Owing to the fact that certain of these data had already apparently been published previously, the Editor of *Experimental and Therapeutic Medicine* has decided that this paper should be retracted from the Journal. The authors were asked for an explanation to account for these concerns, but the Editorial Office did not receive a reply. The Editor apologizes to the readership for any inconvenience caused.

